# A Device for measuring the *in-situ* response of Human Bronchial Epithelial Cells to airborne environmental agents

**DOI:** 10.1038/s41598-019-43784-5

**Published:** 2019-05-13

**Authors:** Lakshmana D. Chandrala, Nima Afshar-Mohajer, Kristine Nishida, Yury Ronzhes, Venkataramana K. Sidhaye, Kirsten Koehler, Joseph Katz

**Affiliations:** 1 Department of Mechanical Engineering, Johns Hopkins University, Baltimore, 21218 USA; 2 Department of Environmental Health and Engineering, Johns Hopkins Bloomberg School of Public Health, Baltimore, 21205 USA; 3 Division of Pulmonary and Critical Care Medicine, School of Medicine, Johns Hopkins University, Baltimore, 21205 USA

**Keywords:** Molecular medicine, Microscopy

## Abstract

Measuring the time evolution of response of Normal Human Bronchial Epithelial (NHBE) cells to aerosols is essential for understanding the pathogenesis of airway disease. This study introduces a novel Real-Time Examination of Cell Exposure (RTECE) system, which enables direct *in situ* assessment of functional responses of the cell culture during and following exposure to environmental agents. Included are cell morphology, migration, and specialised responses, such as ciliary beat frequency (CBF). Utilising annular nozzles for aerosol injection and installing windows above and below the culture, the cells can be illuminated and examined during exposure. The performance of RTECE is compared to that of the commercial Vitrocell by exposing NHBE cells to cigarette smoke. Both systems show the same mass deposition and similar trends in smoke-induced changes to monolayer permeability, CBF and transepithelial resistance. *In situ* measurements performed during and after two exposures to smoke show that the CBF decreases gradually during both exposures, recovering after the first, but decreasing sharply after the second. Using Particle image velocimetry, the cell motions are monitored for twelve hours. Exposure to smoke increases the spatially-averaged cell velocity by an order of magnitude. The relative motion between cells peaks shortly after each exposure, but remains elevated and even increases further several hours later.

## Introduction

Environmental exposure to airborne particulate matter has been associated with a variety of long-term respiratory diseases^1–4^. These studies typically involve either epidemiology, which usually requires years of patient data, or toxicology conducted *in vivo* or *in vitro*. Repeatable and controllable *in vitro* exposures of tissues/cell cultures to aerosols are typically aimed at elucidating the pathways and molecular mechanisms involved^5,6^. Several approaches have been developed to study the biological effect of aerosols on airway epithelium^7,8^. In some *in vitro* studies, the particulate matter is collected on filters and suspended/dissolved in a liquid, where it is exposed to the cells for biological responses^9–11^, a strategy more suitable for cells in liquid culture. However, cells cultured on an air-liquid interface, which emulates *in vivo* exposures, can be exposed to particles in an exposure chamber^8,12,13^. In this case, the cells are cultured on semipermeable inserts which allow for exposure to the aerosols on the apical side of the cell surface while being supplied with culture media from below. A complete direct exposure system generally consists of an aerosol generation and dilution unit, and an exposure chamber, which houses the cell culture. Several such systems have been developed and standardised over the years for toxicity testing of airborne particles^7,14^. These systems employ different mechanisms for aerosol delivery, including diffusion and impaction^15–17^, gravitational cloud settling for liquid aerosols^18^, and electrostatic deposition for charged particles^19^. They are commonly used for studying the effect of cigarette smoke but can be adapted for other aerosols. Several commercial systems are available such as Vitrocell^®^ (Vitrocell^®^ Systems, Waldkirch, Germany)^20–22^, CULTEX^®^ (CULTEX^®^ Laboratories Hannover, Germany)^8^, and British American Tobacco (BAT) exposure chamber (Curbridge Engineering, Southampton, UK)^23^. However, a significant limitation of these systems is that they do not permit direct observation on the cells during the exposure and the cells have to be removed from the chamber for microscopic observations for, e.g., Ciliary Beat Frequency (CBF) measurements. The act of removing and transporting the cells may cause an undesired stress^24,25^. Hence, despite considerable efforts, little is known about the biological responses of epithelial cells to cigarette smoke during the exposure^26,27^. If live imaging were possible, it would have enabled researchers to assess the *time evolution* of biological changes occurring *during* the exposure, including cell morphology, CBF and cell migration.

Cell migration is important in many biological processes, including, e.g., wound healing^28^ and cancer progression^29^. It has been already established that the collective motion of cells in a monolayer is controlled by mechanical and chemical cell-to-cell signaling^29,30^. In previous studies involving Madin–Darby canine kidney cells, the relative motions have been measured under a microscope to characterise the progression of a model wound in an epithelial monolayer^31^. They show that the cells migrate collectively towards the center of the wound to form a confluent layer. Once this monolayer is estabished, the migration diminishes owing to the maturation of cell-cell and cell-substrate contacts^32^. However, external factors, such as viruses, allergens, and pollutants might trigger collective cell motions^33^. Hence, it would be of interest to observe whether cigerette smoke triggers cell motions during and after the exposures.

The present study introduces a novel exposure chamber for Real-Time Examination of Cell Exposure (RTECE) that enables *in-situ* live imaging of cell cultures while being exposed to airborne materials under controlled conditions. The unique features of the system include an optimised nozzle for uniform deposition of aerosols on the cell culture surface, adaptable design to house different cell culture inserts, disposable 3D-printed nozzles to avoid cross-contamination between the experiments, and *in-situ* imaging of cells involving different illumination and microscopic observation techniques. Consequently, the cell culture can be observed continuously for long periods, allowing measurements of the time evolution of CBF, cell migration, and culture morphology. To demonstrate the capability of this system, a confluent monolayer of Normal Human Bronchial Epithelial (NHBE) cells are subjected to two exposures separated by a rest period of 60 min, each consisting of two cigarettes. Initially, the mass deposition, cell permeability, and CBF are compared to those obtained from the Vitrocell^22,34,35^ using the same data collection and analysis procedures. Results show a good agreement. Next, the unique features of RTECE are demonstrated by measuring the time evolution of CBF continuously while maintaining the controlled exposure conditions. The data reveals that the CBF decreases gradually during both exposures. It recovers back after the first exposure, but not after the second one. A second series of experiments uses Particle Image Velocimetry (PIV) to examine the cell motion for 12 hours. It shows that exposure to cigarette smoke increases the spatially-averaged cell velocity by an order of magnitude. The relative motion between cells peaks shortly after the exposures, but remains elevated and even increases further several hours later. These phenomena do not occur for the control case.

## Description of the Exposure System

**Figure 1:**
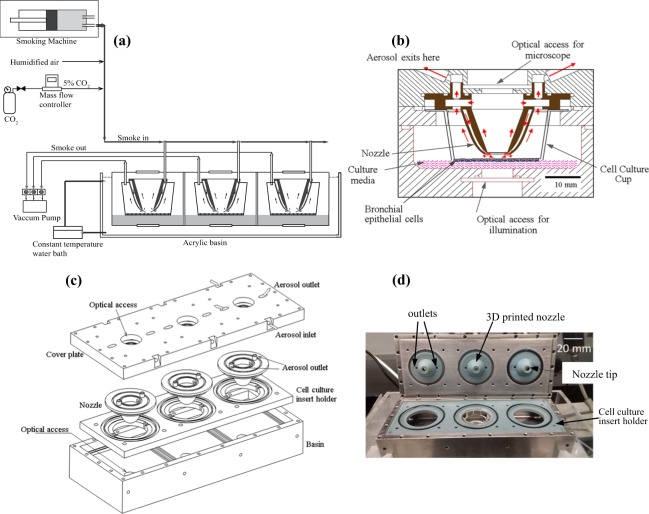
The system for real-time exposure and imaging and of cell cultures to cigarette smoke. (**a**) Overview of the experimental setup. (**b**) Details of the exposure module showing the flow directions. The out-of-plane inlet channels are not visible in this view, (**c**) Components of the system showing how they fit together, and (**d**) an image of the assembled chamber.

A functional schematic of the exposure system is presented in Fig. 1a. It includes a smoking machine for the present application, as well as sources of regulated CO_2_ and humidified air needed for supporting the cell culture. Details about these components are described in the methods section. After mixing the smoke with CO_2_ and humidified air, they are introduced into the exposure chamber, which presently contains three independent modules, via a distribution manifold. Each of the three modules is independently sealed, and could if needed, contain different cultures or exposed differently. The flow is regulated by a vacuum pump located downstream of this chamber. As illustrated in Fig. 1b, the mixture is directed to the cell culture by a custom-made annular nozzle connected to out-of-plane channels, which are not visible in this cross-sectional view. The cell culture is grown on a standard semi-permeable layer on the bottom of a commercial cup and supported by culture media. The gas and particles flow out of the chamber through both the center and perimeter of the nozzle to distribute the particles uniformly. The annular nozzle allows direct microscopic observations on the central 6 mm of the culture, while the exit from the nozzle is located at the periphery of this central zone. In the present setup, the distance between the culture and the upper window is 30 mm, allowing observations using a 20x long working distance objective. Shorter distances and higher magnifications are feasible for future modifications. The bottom of the chamber has another window for illumination. The chamber is placed inside an acrylic basin containing water, which circulated through a heater to maintain a constant temperature of 37 °C.

Figure 1c shows that the exposure chamber consists of four parts, namely, a cover plate, nozzles, holder for the cell culture and nozzles, and the bottom basin containing the culture media. Each is described briefly here. The cover plate and bottom basin are made of stainless steel, facilitating autoclaving, with optical windows for *in situ* imaging. The top plate also contains three independent inlets, which are connected to the distribution manifold, and the outlet ports, which lead to the vacuum pump. It also contains the channel connecting the 3D inlets to the nozzles, and outlet channels leading out from each unit. The flow rate in each unit is adjustable by valves located upstream of the pump. The nozzle and nozzle holder are 3D-printed from Bluestone™ (Accura®) and can be readily re-manufactured. Bluestone (3D Systems Inc.) is a widely used nanocomposite material in Stereolithographic (SLA) printing and has outstanding heat and chemical resistance along with a low cost. For example, it could handle alcohols, and has a heat deflection temperature of 267–284 °C. The nozzles are disposable (along with the cell culture inserts) to prevent cross-contamination between experiments. Inside each unit, the aerosol is injected on the culture by the annular nozzle that has a diameter of 16 mm, an opening of 1 mm, and exit located 3 mm above the culture. The initially-used straight conical nozzles have generated an annular pattern of particle deposition. Consequently, it has been replaced by the current curved annular nozzle that has an exit aimed towards the center of the culture, which achieves a much more uniform deposition. The aerosol stream bifurcates downstream of the nozzle tip. Part of the gas stream flows radially inward and is collected through the central opening of the nozzle. The rest flows radially outward and is collected from the outer perimeter.

**Figure 2:**
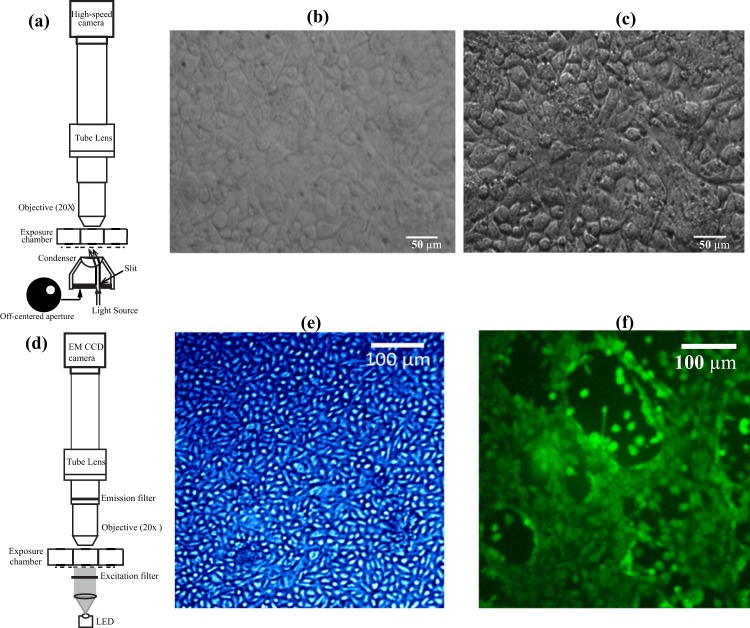
Microscopic images of the Human bronchial epithelial (HBE) cells exposed to clean air. (**a**) Optical setup for oblique illumination microscopy. **(b**) Image obtained by axial illumination, and (**c**) oblique illumination. (**d**) Optical setup for fluorescence imaging. (**e**) Cell nuclei stained by Hoechst 33342, and (**f**) Cell membrane is stained by Calcein.

Two optical modalities, oblique illumination microscopy (Fig. 2a) and fluorescence imaging (Fig. 2d), are employed to visualise the cell culture *in situ*. Oblique illumination microscopy^36^ is used for monitoring the cilia motion and cell migration, and fluorescence microscopy is used for observing fluorescently-stained cells. Each utilises a different camera and illumination source. The oblique illumination is aimed at improving the contrast of images when the sample is illuminated by white light by preventing the forward-scattered light from reaching the imaging system, similar to contrast microscopy. A comparison between images of NHBE cells obtained using axial and oblique illuminations of the same sample exposed to clean air is provided in Fig. 2b,c). The obliquely illuminated culture allows for clearer demarcations of cell-cell boundaries. As discussed later, using a high-speed camera to record the images, the setup in Fig. 1a is used for acquiring the data needed to measure the CBF and cell migration during the present experiments. In addition, to demonstrate the capability to acquire images of fluorescently-labeled cells, we use the forward-illumination configuration shown in Fig. 2d, which includes an LED with the proper wavelength and a narrow-line band-pass filter. In this case, the images are recorded by an EMCCD camera. Figure 2e,f provide two sample fluorescent images of the NHBE cells, the first after staining the nuclei, and the second, after a cytosolic fluorescent marker. As is evident, both the nuclei and cell bodies are distinguishable. Details about the camera models, objectives, light sources, and cell-stains are provided in the methods section.

## Results

### Comparisons between the RTECE and Vitrocell data

**Figure 3:**
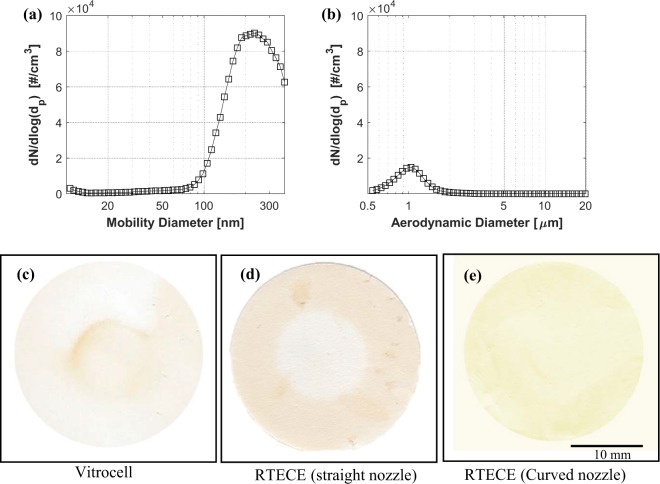
Cigarette smoke particle size and spatial distributions. (**a**) Nano-scale range measured by SMPS, and (**b**) Micro-scale range measured by APS. (**c**–**e**) Spatial distribution of particulate smoke collected on filters placed instead of the culture inside the (**c**) Vitrocell, (**d**) RTECE with the straight conical nozzle, and (**e**) RTECE with the present curved nozzles.

The comparison between the RTECE and Vitrocell systems^22^ is based on exposing cells in each system to smoke generated by four standardised research grade cigarettes (3R4F, University of Kentucky). The size distributions of nano- and micron-sized smoke particles upstream of the instruments have been measured using particle sizing instruments. In the nanoscale range, the size distribution presented in Fig. 3a has a mode at 220 nm mode, consistent with the previous studies^37^. In the micron-scale range, the distribution in Fig. 3b has a single mode at 1 µm. Our first step is a comparison between the mass distribution on the culture in the RTECE and Vitrocell systems. A 25-mm glass fiber filter is placed in the cell culture cups instead of the culture in both devices and exposed to four cigarettes. First, the filters are scanned and their images are compared to determine the spatial distributions of deposition. Two types of annular nozzles with the same outlet diameter are tested in the RTECE, namely one with a straight conical exit, and the other with a curved outlet pointing to the center (Fig. 1b). A standard trumpet-shaped nozzle is used in the Vitrocell system. Sample images of the deposits on the filters are shown in Fig. 3c–e. As is evident, in the Vitrocell (Fig. 3c), most of the mass is concentrated in the center of the filter. In the RTECE with a straight nozzle, most of the mass is deposited along the perimeter of the filter (Fig. 3d). These observations have led us to modify the nozzle geometry and direct the inflow towards the center. The resulting distribution of deposited mass (Fig. 3e) is considerably more uniform than that those of the other cases.

**Figure 4:**
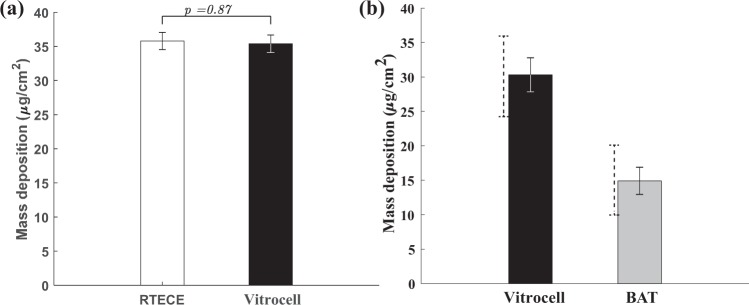
(**a**) Comparison of the present mass deposition in the RTECE (curved nozzle) and Vitrocell systems after exposure to four cigarettes, with error bars denoting the standard deviations among six replicates. The numbers over each bar show the corresponding *p* values. (**b**) Comparison of the published mass depositions in Vitocell^22,34^ to that in the British American Tobacco (BAT) exposure chamber^34,38–40^. Solid error bars represent standard deviations among replicates and different sources, and dashed bars indicate the extent of values involved.

Next, the mass of the filter is measured by an analytical microbalance after exposing to smoke in the Vitrocell and RTECE with the curved annular nozzle. Based on six replicated measurements, the masses deposited in the Vitrocell and RTECE are 35.78 ± 2.15 µg/cm^2^ and 35.37 ± 2.2 µg/cm^2^, respectively, with the uncertainty indicated by the standard deviation among replicates. Figure 4a also provides the results of the direct comparison between the RTECE and Vitrocell mass depositions, showing that they are very similar. In addition, the mass deposition data in the Vitrocell and another device, the BAT exposure chamber is taken from the published literature, and compiled it into a comparison between them. Figure 4b compares the published data for mass deposition in the Virtrocell^22,34^ and BAT^34,38–40^, including results of a direct comparison^34^. Overall, the average Virtocell mass deposition is higher than the compiled average by 50%. However, the present data fall within the range of previously published results, as indicated by the dashed error bars. In contrast, while results vary among the different sources, the mass deposition per unit area in the BAT is persistently lower than that in the Vitrocell.

**Figure 5:**
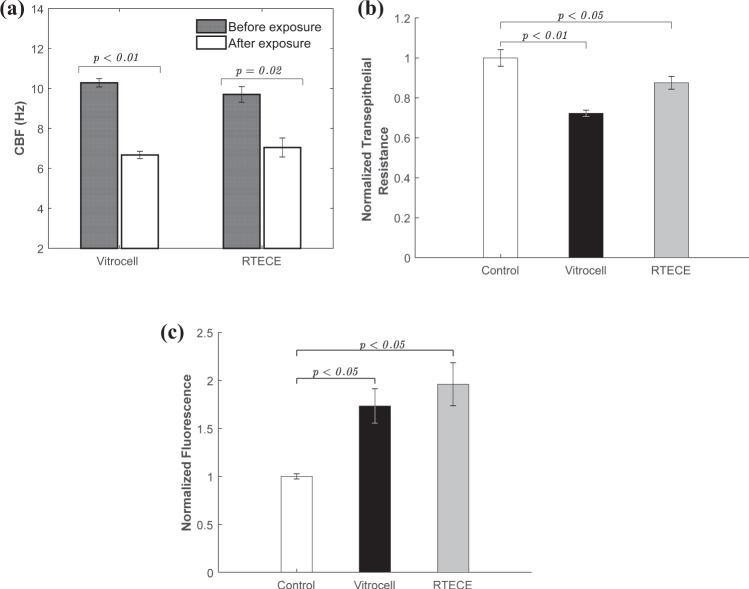
Comparison between the biological responses of Normal human bronchial epithelial cells exposed to cigarette smoke in the Vitrocell and the RTECE. (**a**) Cilia Beating Frequency, (**b**) Normalized Transepithelial resistance and, (**c**) Epithelial monolayer permeability, as measured by FITC-dextran assays. Control case represents exposure to clean air. Error bars denote the standard error among three replicates. The numbers over each bar show the corresponding *p* values.

Figure 5a shows the effect of exposure to cigarette smoke on the CBF of similar cultures in the RTECE and Vitrocell systems. In both cases, the CBF is measured before and two hours after two exposures by removing them from their respective chambers and observing them under a microscope. Using procedures described in a previous study^19^, the CBF is determined based on spectral analysis of time series of intensity in each pixel of the image, and averaging the results for the entire field of view. The results based on three replicates are summarised in Fig. 5a. Samples removed from the Vitrocell and RTECE systems show similar trends with no significant difference (*p* = 0.45) between them. Both indicate a significant decrease (*p* < 0.05) in CBF two hours after exposure to smoke, consistent with the previous studies^41^. Results for the Transelectrical epithelial resistance (TEER) of the culture after exposure to cigarette smoke are compared to a control case in Fig. 5b. Both samples show a decrease in resistance compared to the control case, indicating a decrease in epithelial barrier function^42^, with the Vitrocell culture experiencing a larger decrease. The paracellular permeability of the cell monolayer post exposure, evaluated based on the 4kD FITC dextran permeability, is presented in Fig. 4c. Replicated samples removed from both the RTECE and Vitrocell show a significant increase (*p* < 0.05) in permeability, which indicate a decrease in cell monolayer integrity^43^. The RTECE results have higher averaged values, but the difference between them does not deviate beyond the variability among replicates. In summary, trends in the biological responses to smoke of cultures removed from the Vitrocell and RTECE, evaluated based on CBF, TEER, and permeability show similar trends. These findings support the use of the RTECE system for *in vitro* studies while observing the cell cultures without having to remove it from the exposure chamber.

### *In situ* measurements of CBF and Cell migration during exposure

**Figure 6:**
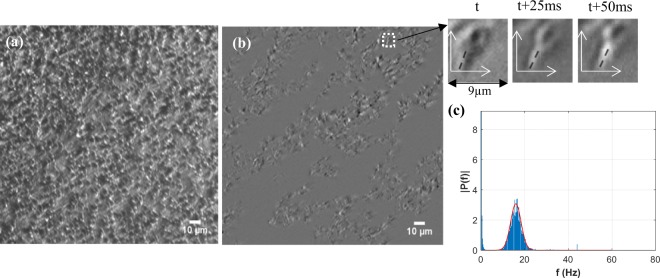
Real-time measurements of ciliary beating frequency in the RTECE. (**a**) Raw image, (**b**) the same image after high-pass filtering, with insets showing time variations in the location of a single cilium, and (**c**) Temporal spectra of intensities in each pixel based on analysis of 512 images acquired at 160 frames per second. The gaussian fit is used for determining the mean CBF.

The RTECE system enables continuous monitoring of the time-evolution of CBF while maintaining the cultures in the controlled environment. Prior to exposure, the cells are maintained in the chamber for 30 min while injecting clean air at 37 °C, 5% CO_2_ concentration, and 95% relative humidity. Figure 6a shows a sample image obtained during exposure to cigarette smoke, focusing on a plane located about 1μm above the NHBE cells to observe the cilia motion. To demonstrate the ability to detect the cilia, the time-series of the intensity of each pixel is high-pass filtered at a frequency of 6 Hz, which removes the background intensity variations. The resulting image in Fig. 6b (the corresponding movie is shown in Supplementary Video S1), along with insets containing a magnified section of 30 × 30 pixels at three times (Δ*t* = 25 ms, *t* being time), confirms that the motion of cilia can be readily detected. To determine the CBF, Fast-Fourier Transform (FFT) is used for calculating the temporal spectrum of the intensity in each pixel in the original unfiltered images. Results for the entire field of view are then averaged to obtain the CBF. Figure 5c shows a sample temporal spectrum containing the FFT measured by analysing each pixel in a sequence of 512, 2048 × 1088 pixels images acquired at 160 frames per second during a period of 3.2 s. A Gaussian fit to the data is used for determining the mean value. During the test, this procedure is repeated every 5 min for ~2.5 hours for the control and exposure cases.

**Figure 7:**
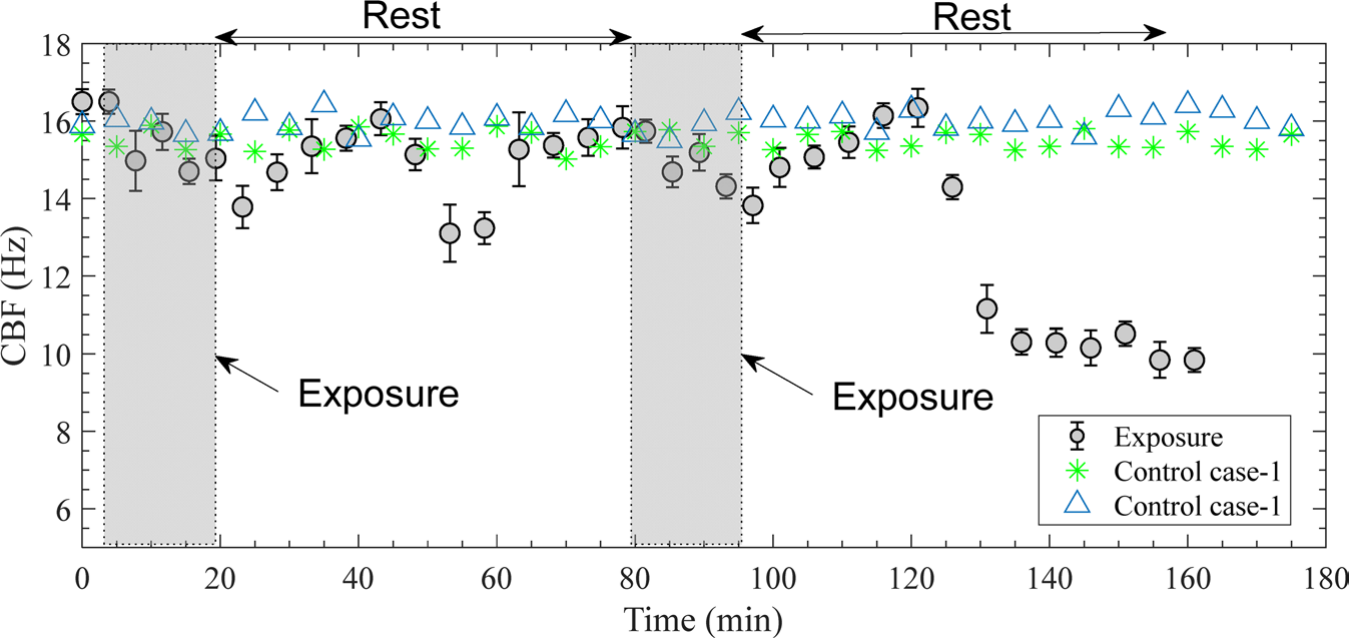
Time evolution in ciliary beat frequency of human bronchial epithelial cells during and after exposures to cigarette smoke (shown by circles). Error bars denote the standard error among three replicates. Control cases are representing exposure to clean air. Each case is replicated twice with results shown separately.

Figure 7 shows the time evolution of CBF during and after exposure to cigarette smoke along with two cases of control data. As is evident, there are small differences between the two control cases, and both show little differences in CBF for the entire duration of the experiment. In contrast, the smoke causes a gradual decrease in CBF that starts immediately and persists for the entire 16 min of exposure and 4 min afterwards, decreasing from 16.5 to 13.8 Hz (*p* < 0.01). During the first rest period, the CBF fluctuates, increasing for the first 20 min, then decreasing for 10 min, but recovering back to slightly below the initial value at the end of the 60-minute rest period. A similar gradual decrease in CBF occurs during the second exposure, but after a ~24 min recovery period to the initial level, the CBF decreases rapidly to about 10 Hz and does not recover for at least one hour after the second exposure. Results for the three replicates show very similar trends (*p* = 0.92). In summary, the time evolution of CBF in the RTECE chamber indicates that the cell culture recovers after the first exposure to two cigarettes. While it initially recovers after the second exposure as well, it subsequently fails with the CBF decreasing from 16.3 to 10 Hz.

**Figure 8:**
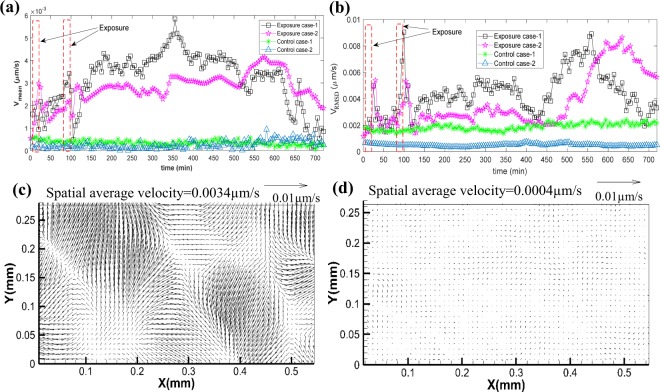
Exposure to cigarette smoke increases the motion of human epithelial cells. (**a**) Time evolution of spatially-averaged cell velocities during and after exposure to cigarette smoke is compared to those of control (clean air) cases. (**b**) Comparisons of the corresponding spatial RMS of the cell velocity. Each case is replicated twice with results shown separately. Sample instantaneous deviations from the spatially-averaged velocity for the (**c**) cigarette exposure, and (**d**) control cases.

Another component in the dynamics of the NHBE response to smoke involves confluent migration of the culture and motion of the cells relative to each other within the cuture. In the observations aimed at measuring the cell migration, the contrast images are acquired every five minutes for 12 hours. As described in the Methods section, particle image velocimetry is used for determining the two-dimensional velocity distributions from the displacement of the cells at a spatial resolution of 3.2μm (~ cell size). The time-varying spatially averaged velocity of the cells is recorded. Then, by calculating the square root of the squared difference between the local and spatially averaged velocity magnitude, one can obtain the spatial Root Mean Square Deviation (RMSD) of cell velocity. Its magnitude is a measure of the relative motion between cells. The tests are replicated twice for the control and exposure cases, and results are shown separately in Fig. 8. Sample movies showing the time evolution of cell motions for one of the exposures (Supplementary Video S2) and control cases (Supplementary Video S3), as well as the velocity distributions, with (Supplementary Video S4) and without (Supplementary Video S5) subtracting the spatially averaged velocity for one of the exposure cases, are provided as supplemental information.

Figure 8a,b show the time evolution of the spatially-averaged and spatial RMSD values of cell velocity for the control and exposure cases, respectively. Sample vector maps of the instantaneous deviations from the spatially-averaged velocity for the cigarette exposure and control cases are provided in Fig. 8c,d, respectively. Reference vectors are provided above each plot. The first vector map (Fig. 8c) is a representative for high spatial RMSD value during exposure to smoke indicating high relative motions between cells, and the latter is a representative low RMSD level for one of the control cases. As evident from Fig. 8a, the average velocity of cells in both control cultures remains persistently low (<0.5 × 10^−3^ μm/s) for the entire 12 hours of exposure. The spatial RMSD values of the two control cases have different values, but both remain constant and low for the entire duration of the experiment. In contrast, exposure to cigarette smoke generates considerable motion and spatial variability in cell velocity. The spatially-averaged velocity increases during the first exposure, and fluctuates shortly afterwards while gradually increasing. There is a sharper increase during the second exposure, followed by a rapid decrease shortly afterwards, and a subsequent persistent increase to a level ranging from 2.5 × 10^−3^ to 5 × 10^−3^ μm/s for more than 8 hrs after the second exposure. Only later, the average velocity begins to decrease while still fluctuating. The spatial RMSD values of both exposure cases have sharp peaks shortly after the exposures. For the next 4.5 hours, the RMSD levels remain elevated, and then decrease to the control level after about 5 hrs. Subsequently, in the absence of additional stimuli, both RMSD values rize substantially, peaking 6–8 hrs after the second exposure, and then decrease again. Clearly, the *in situ* observations in the RTECE system demonstrate that exposure to cigarette smoke causes a substantial increase in the average velocity and relative motion among cells that persist for many hours. To the best of our knowledge, such trends have never been seen before and require future characterisation. Yet, the following discussion mentions other studies, which do not involve smoking, where the relative motion of cells within cultures have been measured under a microscope to characterise the progression of wounds^28,29^.

## Discussion

The present study introduces a novel exposure chamber for direct *in-situ* observations on the time evolution of cell cultures during exposure to airborne particles. By using annular nozzles for injecting the aerosol and installing windows above and below the culture, it can be illuminated and examined during the exposure. In contrast, existing commercially-available systems, such as Vitrocell^®^ and CULTEX^®^, do not have optical access^7,14^, requiring removal of the cells from the chamber for microscopic analysis. The new RTECE setup consists of three independently sealed chambers submerged in a constant temperature water bath. These chambers could be operated (and examined) in parallel. The annular nozzles are disposable, and their geometry could be modified, e.g., reduced in size to accommodate smaller cell culture inserts. Being disposable reduces the likelihood of cross-contamination, and by 3D printing the nozzle from Bluestone™ (Accura®) using widely-available printers, the associated costs are insignificant. The total cost of fabricating the RTECE chamber is about $2,500 (not including engineering costs). The optical components of the microscopy system including 170 frames per second camera, objectives, optical mounts, light sources, windows and filters are approximately $9,000. The only non-dedicated expensive component is the EMCCD camera used for low-light-levels fluorescence microscopy ($35,000). This camera is only used for acquiring the images in Fig. 2e,f. The present distance between the culture and the observation window restricts the magnification to 20x, but the nozzle and chamber could be redesigned to allow higher magnifications. Furthermore, the optical setup is not restricted to the demonstrated fluorescence imaging and oblique illumination microscopy^36^. Other techniques, such as Differential Interference Contrast^44^ microscopy, could also be utilised. While this paper focuses on response to cigarette smoke, the RTECE system could be readily connected to other aerosol generators, e.g. a Collision Nebulizer (BGI, Mesa Labs Inc.,) or Small-Scale Powder Disperser (SSPD, Model 3343, TSI Inc.,).

The response of cell cultures to cigarette smoke in the RTECE and Vitrocell^®^ systems are compared by measuring the mass deposition, CBF, as well as transepithelial resistance and permeability of cell cultures, following the same data collection and analysis procedures. The differences in mass deposition are insignificant. By aligning the RTECE nozzle exit towards the center of the culture and forcing the jet to flow both radially inward and outward, appears to homogenise the spatial distribution of mass deposition. The smoke causes a reduction in CBF and an increase in permeability of both the RTECE and Vitrocell^®^ cultures, with the differences between them falling within the standard error of three replicates. Cultures exposed in both systems also show a reduction in transepithelial resistance with low standard errors, but the RTECE values after exposure are higher. These agreements support the further application of the RTECE system for *in vitro* studies.

A natural next step involves *in situ* measurements of the time evolution of CBF since prior research involving cultures removed from the chamber has shown that the CBF decreases after cigarette exposure^42,45^. The continuously-recorded data is obtained while maintaining controlled conditions, consisting of two exposures to two cigarettes each, separated by a rest period of 60 min. The CBF decreases gradually during both exposures, followed by a period of recovery back to the original level about 25 min later. Subsequently, while it fluctuates and eventually recovers after the first exposure, the CBF decreases sharply and fails to recover after the second exposure. In contrast, the frequency of the control cases does not change significantly for the entire three-hour observation period. These findings demonstrate a complex, but repeatable time evolution of response, which depends on the number of exposures, and presumably, the length of the rest period. To the best of our knowledge, such fluctuations in CBF have never been shown before.

Time-lapsed observations for twelve hours are used for examining the confluent migration of the culture and motion of the cells relative to each other. As noted before, such migrations have been linked to wounds, as well as exposure to viruses, allergens, and pollutants in observations performed under a microscope^31,33^. In agreement with previous observations^33^, in the present conrol cases, the spatially averaged migration speed is very low (<0.5 × 10^−3^ μm/s). The corresponding spatial RMSD values, represting the relative motions, have different magnitudes, but both remain low and nearly constant. In contrast, exposure to cigarette smoke triggers confluent migration of the cell culture at a speed that is at least an order of magnitude higher than the control that persists, with varying magnitudes, for the entire duration of the tests. Furthermore, the RMSD level peaks briefly after each exposure and then decreases again, but remins elevated. Then, 6–7 hours after the second exposure, the relative motions rise again and remain high for about two hours. Interestingly, at about 135 min, when the CBF decreases sharply, the confluent velocity is rising rapidly, while the RMSD level is low. While the cell mechanical and bio-chemical causes for these motions and their correlation to the CBF are beyond the scope of the present observations, the RTECE system enables us to demonstrate that the cigarette smoke affects the culture dynamics for many hours after the expoure. Future studies will focus on the associated mechanisms.

## Methods

The RTECE system is illustrated in Fig. 1 and introduced in the main text. This section provides supporting technical information about the preparation of cell cultures, test conditions, data acquisition and analysis procedures, and peripheral instrumentation.

### Cigarette smoke generation and monitoring

The exposure system (Fig. 1a) includes a manual smoking machine (Model VC1, VitroCell^®^ Systems), as well as sources of regulated CO_2_ and humidified air needed for supporting the cell culture. The smoking machine consists of a lighting chamber, in which the cigarettes are loaded one-by-one, and a piston pump that samples a prescribed volume of smoke and forces it into the exposure chamber. In the present study, the machine is configured to generate 35 mL puffs, each with a duration of 2 s, once in every 60 s. These specifications comply with the International Organization for Standardization (ISO) 3308 puff profile. Each cigarette (3R4F, Research Cigarette, University of Kentucky) is puffed eight times over a period of 8 min, and each exposure consists of two cigarettes. In all the experiments, cells are subjected to two exposures, separated by a rest period of 60 min. The smoke is mixed with humidified HEPA-filtered air containing 5% by volume CO_2_ and directed to the distribution manifold using Teflon tubes. Humidification is performed by passing 1 LPM of the HEPA-filtered air through a 500-mL bubbler bottle (Schott Duran). The Relative Humidity (RH) is maintained at 95–96%, as measured before each experiment by a real-time hygrometer (Fisherbrand™, Fisher Scientific). The CO_2_ flux is regulated by a mass flow controller (MC-100SCCM, Alicat Scientific). The flow to all the test chambers is regulated by a vacuum pump (Model N 86 KTP, KNF Neuberger Inc), mass flow meter (GFMS-010449, Analyt-MTC) and valves located downstream of the RTECE system. The flow rate into each unit of the exposure chamber is maintained at 5 mL per minute, and the rest of the flow is released to the atmosphere through the bypass line containing a one-way check valve. The same approach is used for driving and regulating the flow into the Vitrocell.

To obtain the concentration and particle size distributions of the cigarette smoke, the aerosol generated by the smoking machine is diverted into a 25 × 15 × 25 cm^3^ acrylic chamber connected to two particle sizing instruments. The nano-sized particles (10–370 nm) are measured using a Scanning Mobility Particle Sizer, SMPS Model 3938 (TSI), with an electrostatic classifier model 3082, and a particle counter model 3787. An impactor with an orifice diameter of 0.071 cm is installed at the inlet of the classifier to remove particles larger than 500 nm. The micron-sized particles (0.5–20 µm) are sampled using an Aerodynamic Particle Sizer, APS model 3321 (TSI). Samples are collected for 32 min while the smoke generator is working, and then averaged.

### Preparing the bronchial epithelial cell culture

Primary NHBE cells (MatTek corp) have been grown on collagen-coated transwell inserts (Coring®) immersed in bronchial epithelial growth media (Lonza, Stemcell) at 37 °C and 5% CO_2_. Once the cells form a confluent monolayer, they are transferred from a submerged condition to an air-liquid interface (ALI) condition by removing the apical media. The cells are maintained at the ALI condition for 4–6 weeks, at which time they are terminally differentiated and comprised of clara, basal, goblet, and ciliated cells forming a tight monolayer, as described in the literature^46,47^. The resulting culture is then inserted in the exposure chamber while maintaining an ALI condition.

### Imaging set-up

The optical set-up for white-light oblique illumination is shown in Fig. 2a. The output of a collimated halogen lamp (Model U-LH100-3, Olympus corp.), located under the camber, is focused by a condenser lens with a working distance of 40 mm on the cell culture. The oblique illumination^36^ is achieved by placing an off-centered aperture before the condenser lens. A 20x infinity-corrected long-working distance objective (Mitutoyo) combined with a 200 mm focal length tube lens (Edmund Optics) is used for magnifying the images. This objective has a working distance of 34 mm needed for focusing on the cell culture inside the exposure chamber. The images are acquired by a high-speed camera (Model Lt225, Lumenera Corp.) that has a 2048 × 1088 pixels CMOS sensor and pixel size of 5.5 𝜇m. For measuring the CBF, the sampling rate is 160 frames per second (fps), each with an exposure time of 3 ms. For measuring the cell migration, isolated images are recorded every 5 min. In both cases, the field of view (FOV) is 552 × 293 𝜇m^2^.

The optical configuration for fluorescence imaging is shown in Fig. 3b. Cell cultures labeled with nucleus stain (Hoechst 33342) and cell membrane stain (Calcein AM) are illuminated from below by collimated LED beams with peak wavelengths 380 nm and 490 nm, respectively. The excitation and emission bandpass filters for nucleus stain are centered at 380 nm (bandwidth of 30 nm) and 450 nm (bandwidth of 20 nm), respectively. The corresponding filters for membrane stain are 490 nm (bandwidth of 20 nm) and 520 nm (bandwidth of 10 nm). Using the above-mentioned microscopic objective and tube lens, the low-intensity images are recorded by an EMCCD camera (iXon Ultra 888, Andor) operating at an EM gain of 300. The frame rate of this camera varies from 26 to 697 fps, and the corresponding pixel array sizes are 1024 × 1024 pixels and 128 × 128 pixels.

### Mass deposition measurements

For measuring the particulate mass deposited on the culture, a 25-mm fluorocarbon-coated glass fiber filter (FiberFilm T60A20, Pall Inc.) is placed in the inserts instead of the cell culture. The filters are weighed before and after the exposure using an analytical microbalance, model MX5 (Mettler Toledo), at a precision of 1 µg to determine the net mass deposited on them. Before weighing the filters, they are equilibrated in a clean room under controlled temperature and relative humidity (T = 21 ± 3 °C and RH = 30 ± 2%) for at least four hours, following the National Institute for Occupational Safety and Health (NIOSH)^48^ procedures.

### Transepithelial electrical resistance (TEER) measurements

The TEER of the transwell cultures in ohms is measured using an epithelial volt/ohm meter (EVOM2, World Precision Instruments). An electrode is placed on each apical and basal side of the transwell culture, and an alternating voltage is applied to measure the resistance^49^. Three independent measurements are performed for each insert and averaged to obtain the TEER.

### Monolayer permeability assays

Paracellular permeability is assessed using a 4kD FITC dextran assay (Sigma-Aldrich), following the previous studies^49^. After each exposure test, 2–3 mL of phosphate-buffered saline (PBS) solution is added to the basal compartment of the transwells, and 1–2 mL of 4kD FITC dextran at a concentration of 0.5 mg/mL is added to the apical side of the cells. The cells are then incubated at 37 °C, 5% CO_2_, and 95% RH for 30–45 min. Following incubation, a fluorimeter is used for determining the fluorescence of the sample solution taken from the basolateral compartment.

### Cell migration analysis

Standard Particle Image Velocimetry (PIV)^50^ procedures are used for measuring the two-dimensional velocity distribution from the displacement of the cells in the culture. PIV is a popular and widely used method for mapping the velocity in planar sections of a flow field. Typically, it consists of recording image pairs of a flow field illuminated by a laser sheet and seeded with tracer particles. The images are divided into interrogation windows, and the displacement of the particles in each window is determined by cross-correlating the corresponding intensity distribution in the two images. In the present study, to quantify cell migration, contrast images of the cell culture are acquired every five minutes for 12 hours. Cross-correlation analysis of successive images is then processed in a similar manner to standard PIV using the commercial software package DaVis© (LaVison Inc.). Multi-pass cross-correlation procedures with decreasing interrogation window size are used for optimising the data quality. The final interrogation window size is 64 × 64 pixels, corresponding to 3.2 × 3.2 µm, i.e. about one cell size. With 75% overlap between adjacent windows, the resulting velocity vector spacing is 0.8 × 0.8 µm. The data post-processing includes removal of spurious vectors using universal outlier detection^51^. The spatially-averaged velocity magnitude is calculated by averaging the instantaneous velocity over the entire field of view. It describes the displacement of the entire culture. Then, by squaring the difference between the local and spatially-averaged velocity magnitude, and then averaging the results over the entire field of view, one obtains the spatial Root Mean Square Deviation (RMSD) of velocity. The latter is a measure of the velocity difference between cells in the culture.

## Supplementary information

Supplementary Video 1

Supplementary Video 2

Supplementary Video 3

Supplementary Video 4

Supplementary Video 4

Supplementary Information
