# Supplementary material for: A Device for measuring the in-situ response of Human Bronchial Epithelial Cells to airborne environmental agents
Source: Sci Rep. 2019 May 13;9:7263. doi: 10.1038/s41598-019-43784-5 (PMC6513995; doi:10.1038/s41598-019-43784-5)
Supplement: Supplementary file 6 — Supplementary Information [file 41598_2019_43784_MOESM6_ESM.docx]

**Supplementary information**

**A Device for measuring the *in-situ* response of Human Bronchial Epithelial Cells to airborne environmental agents**

Lakshmana D Chandrala^1^, Nima Afshar-Mohajer^2^, Kristine Nishida^3^, Yury Ronzhes^1^, Venkataramana K. Sidhaye^2,3^, Kirsten Koehler^2^, Joseph Katz^1*^

^1^ Department of Mechanical Engineering, Johns Hopkins University, Baltimore, 21218, USA
^2^ Department of Environmental Health and Engineering, Johns Hopkins Bloomberg School of Public Health, Baltimore, 21205, USA
^3^ Division of Pulmonary and Critical Care Medicine, School of Medicine, Johns Hopkins University, Baltimore, 21205, USA

*Corresponding author: Joseph Katz (katz@jhu.edu)

**Supplementary Video legends**

**Supplementary video S1.** Sample movie showing the time evolution of cilia during the exposure to cigarette smoke. The video is obtained after the high-pass filtering the time-series of the intensity of each pixel is at a frequency of 6 Hz.

**Supplementary video S2.** Sample movie showing the time evolution of cell motions for one of the exposure cases.

**Supplementary video S3**. Sample movie showing the time evolution of cell motions for one of the control cases.

**Supplementary video S4**. Sample movie showing the time evolution of spatially-averaged cell velocities during and after exposure to cigarette smoke.

**Supplementary video S5**. Sample movie showing the time evolution of instantaneous deviations from the spatially-averaged velocity during and after exposure to cigarette smoke.
